# Rapid detection and monitoring of human coronavirus infections

**DOI:** 10.1016/j.nmni.2018.04.007

**Published:** 2018-05-09

**Authors:** A.H.L. Bruning, H. Aatola, H. Toivola, N. Ikonen, C. Savolainen-Kopra, S. Blomqvist, D. Pajkrt, K.C. Wolthers, J.O. Koskinen

**Affiliations:** 1) Department of Medical Microbiology, Academic Medical Center, Amsterdam, The Netherlands; 2) ArcDia International Oy Ltd, Turku, Finland; 3) National Institute for Health and Welfare (THL), Helsinki, Finland; 4) Department of Pediatric Infectious Diseases, Emma Children's Hospital, Academic Medical Center, Amsterdam, The Netherlands

**Keywords:** Human coronavirus, point-of-care test, rapid antigen test, rapid detection, respiratory tract infection

## Abstract

Human coronaviruses (CoVs) are increasingly recognized as important respiratory pathogens associated with a broad range of clinical diseases. We sought to increase the insight into clinically relevant CoV infections by monitoring antigen concentrations in six confirmed CoV-positive patients using a newly developed assay for rapid detection of CoV OC43 infections. Antigen positivity lasted 3 to 6 days in secondary infections and 13 days in primary infection. CoV infections are clinically diverse, are common, and cannot be diagnosed from clinical symptoms alone.

## Introduction

Coronaviruses (CoVs) are large, enveloped, single-stranded, positive-sense RNA viruses that belong to the *Coronaviridae* family. Although the first two human CoVs—CoV-229E and CoV-OC43—had already been discovered in the 1960s, no special attention was given to them because infections were primarily self-limiting and were only associated with symptoms of the mild common cold [1]. Since 2000, several new CoV types have emerged. In 2003, the World Health Organization issued a global alert about a deadly new infectious disease, severe acute respiratory syndrome, which turned out to be caused by a CoV [2]. In late 2004 a novel CoV, NL63, was isolated from two children with respiratory symptoms in the Netherlands, followed by the discovery of CoV-HKU1 in a patient with pneumonia. In 2012, the Middle East respiratory syndrome CoV was identified and was acknowledged to be one of the most dangerous respiratory viruses for humans [3], [4].

As a result, CoVs are increasingly recognized as important pathogens associated with a broad range of clinical diseases. Previous studies have reported CoV-OC43 to be the most prevalent CoV in many countries [5], [6]. Virus isolation in cell culture and more recently molecular techniques, specifically PCR, have been the method of choice for diagnosing CoV infections, but they have several disadvantages [4], [7]. Commercial PCR-based methods are often relatively expensive, they require technical expertise and the presence of viral RNA or DNA does not always reflect acute disease. Moreover, using PCR, CoVs are frequently codetected with other respiratory viruses, and the contribution of positive CoV PCR results to disease severity is not always clear [8], [9].

Despite the high morbidity and mortality associated with infections caused by some specific CoVs and the frequent detection of CoV in patients with respiratory infections, there is no rapid method available that can detect clinically relevant CoVs in humans. The aim of this study was to increase our insight in clinically relevant CoV infections by monitoring antigen concentrations in confirmed CoV patients using a newly developed assay for the rapid detection of CoV-OC43 infections.

An assay to detect species-specific CoV-OC43 nucleoprotein antigens was introduced to the mariPOC respi test in 2017. mariPOC (ArcDia Int. Ltd., Turku, Finland) is an automated and multianalyte antigen detection test system that enables rapid detection of acute infections [10], [11], [12]. Besides the recently added CoV-OC43, the mariPOC respi test is able to detect nine respiratory viruses (influenza A and B viruses, respiratory syncytial virus, adenovirus, human metapneumovirus, parainfluenzavirus type 1–3, human bocavirus) and *Streptococcus pneumoniae* from one nasopharyngeal sample at the point of care. The new CoV antigen test has an analytical sensitivity of 2 ng/mL for OC43 recombinant antigen. The test cross-reacts with neither HKU1, NL63, and 229E nor with other common respiratory pathogens or microbiota. It is therefore unlikely to cross-react either with Middle East respiratory syndrome CoV or severe acute respiratory syndrome CoV. According to the manufacturers' specification, the clinical specificity of the test is 99.4% (n = 160) compared to PCR.

For this study, we used the semiquantitative property of the mariPOC analysis to obtain CoV antigen levels by extrapolation from a standard concentration curve. For verification of the results, samples were sent to two laboratories (Laboratory of Clinical Virology, Academic Medical Center (AMC), The Netherlands; and the National Institute for Health and Welfare (THL), Finland) for PCR testing with a multiplex RT-PCR [13] and a CoV-species–specific RT-PCR [8], respectively.

## Case description

**Fig. 1:**
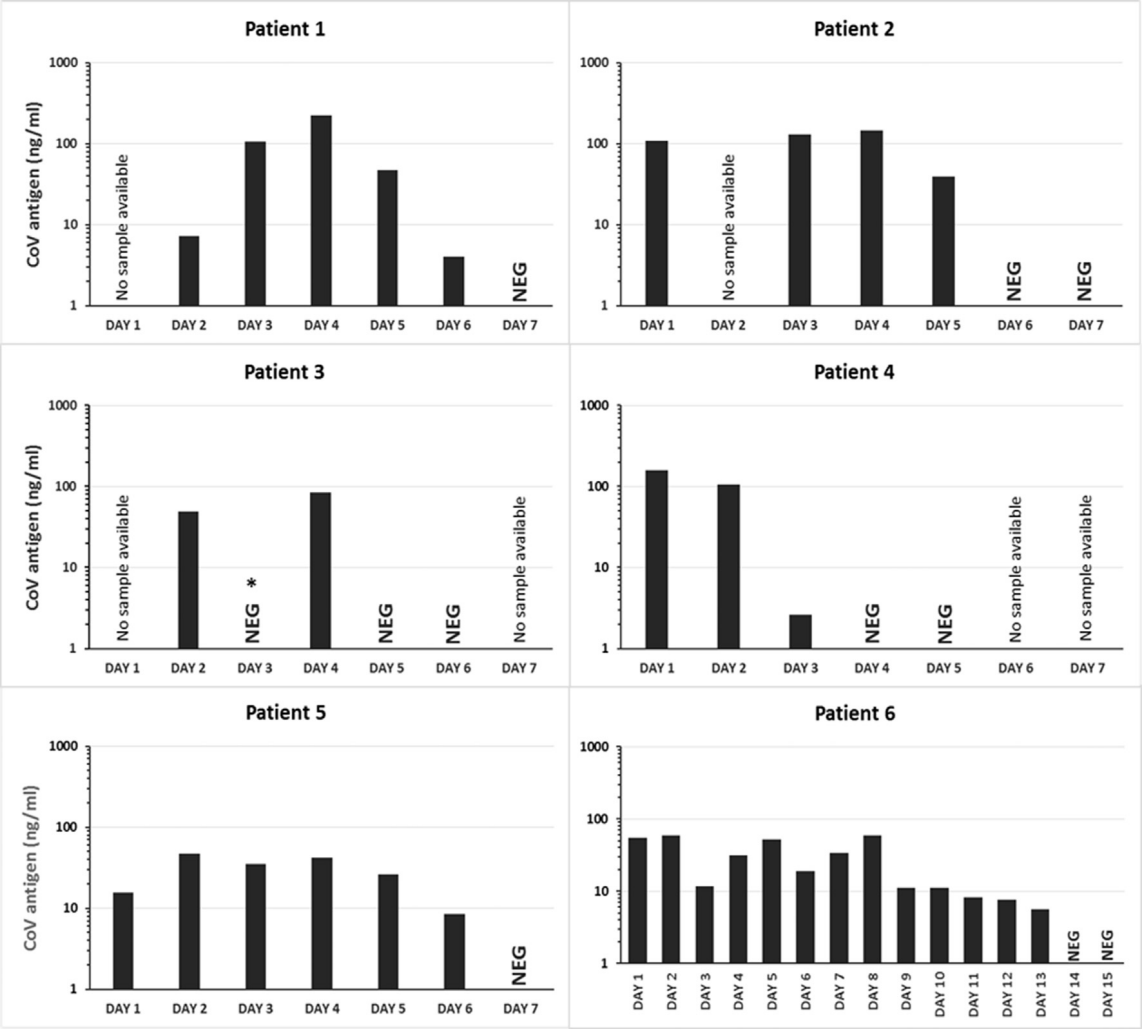
Detection of coronavirus antigen by mariPOC in six patients with respiratory tract infection symptoms. Results are shown from date of symptom onset. Bars marked with ‘NEG’ display samples with a mariPOC signal below cutoff for positive finding. One sample obtained in middle of positivity period (marked with asterisk) was also negative by PCR, suggesting that sample collection was unsuccessful.

We prospectively followed six otherwise healthy immunocompetent Finnish volunteers who developed respiratory illness symptoms and tested positive for CoV-OC43 in the mariPOC assay between December 2015 and December 2016. Informed consent was obtained from patients or their parents before enrolment. After verification of symptoms, nasopharyngeal swabs were collected daily from onset of disease until disappearance of the symptoms. The patients were negative for all other ten pathogens covered by the mariPOC respi test. After mariPOC analysis, all samples were frozen at −20°C, and aliquots were sent to the AMC and THL for confirmation of the results. Antigen measurement results from (almost) daily collected samples are shown in Fig. 1. Antigen secretion correlated relatively well with symptom severity. The clinical characteristics of the CoV-positive patients are provided in Table 1. All samples with measurable CoV antigen levels in mariPOC were also positive by both PCRs.

**Table 1:** Clinical characteristics of coronavirus-positive patients

Patient No.	Sex	Age (years)	Duration of symptoms (days)	Fever	Rhinitis	Cough	Other symptoms
1	F	28	6	Day 1	Days 2 to 6	Days 3 and 4	Fatigue
2	F	28	7	Days 1 to 6	Absent	Days 2 to 4	Headache, fatigue, myalgia, pharyngitis
3	M	46	9	Days 2 to 5	Days 2 to 6	Days 4 to 9	Headache, fatigue, otalgia
4	M	28	4	Absent	Absent	Absent	Headache, fatigue, myalgia
5	M	36	4	Absent	Days 1 to 4	Absent	Fatigue
6	F	2	14	Days 1 to 3	Days 1 to 14	Days 2 to 8	Fatigue

## Discussion

Because of its frequent detection and the potential severe complications associated with CoV infection [14], [15], new diagnostic methods to rapidly identify these infections are needed. With the newly developed CoV antigen assay, we successfully monitored six CoV-positive patients. We showed that CoV infections are clinically diverse and, as also has been shown by earlier studies [16], cannot be diagnosed on the basis of clinical symptoms. Our results suggest that the assay could potentially identify patients in whom CoV is the real cause of the infection because it measures the virus itself, and the antigen level needed for detection is achieved only during the acute phase of the infection, as has also been the case with influenza [17]. However, larger studies with more patients are needed to confirm these findings and to further determine the full diagnostic accuracy of the new assay.

The young age, the more severe illness episode and the long virus positivity time suggests that patient 6 probably had a primary infection. On the basis of patient age and data from seroprevalence studies, the other cases were likely secondary infections. Interestingly, patient 3 was diagnosed as CoV-OC43 positive again 20 months after the infection described above (data not shown), which confirms the widespread prevalence, the possibility of reinfection and the apparent lack of protecting immunity against the same subtype of CoV [18].

Monitoring the antigen concentrations suggested that virus load peaked around the third and fourth day after symptom onset, which confirms the findings in the experimental study by Adney et al. [19]. Sampling should therefore be done within the first 4 days of symptom onset in order to ensure maximum sensitivity of antigen detection testing. The patient with a likely primary infection showed antigen positivity for 13 days, which is about 1 week longer than the positivity times in adults and what is usually observed for other viruses [20]. Prompt testing and diagnosis maximize the potential to affect treatment decisions, such as prescribing virus-specific drugs, predicting the clinical course and withholding prescription of antibiotics. The new rapid test might therefore be a valuable contribution to patient care.
